# Bidirectional predictors between baseline and recovery sleep measures and cardiovascular measures during sleep deprivation and psychological stress

**DOI:** 10.14814/phy2.70374

**Published:** 2025-05-22

**Authors:** Lauren N. Pasetes, Kathleen M. Rosendahl‐Garcia, Namni Goel

**Affiliations:** ^1^ Biological Rhythms Research Laboratory, Department of Psychiatry and Behavioral Sciences Rush University Medical Center Chicago Illinois USA; ^2^ Houston Methodist Academic Institute Houston Texas USA

**Keywords:** biomarkers, cardiovascular, echocardiography, psychological stress, recovery, sleep deprivation

## Abstract

For the first time, we investigated bidirectional predictors between baseline and recovery sleep and cardiovascular (CV) measures during total sleep deprivation (TSD) and psychological stress in a five‐day experiment with 32 healthy adults (27‐53y; 14 females). CV measures were collected in the morning after two baseline nights (B1, B2) and during TSD morning (TSD AM) and evening following psychological stress (TSD PM). Actigraphy assessed sleep during B2 before TSD and the first recovery night (R1) after TSD. Higher B2 wake after sleep onset (WASO) predicted lower TSD PM stroke volume and higher TSD PM systemic vascular resistance index (SVRI), with greater B2 percent sleep predicting inverse relationships, explaining 12.8%–15.9% of the TSD CV variance. Also, higher B2 WASO predicted higher B2 AM SVRI. Furthermore, longer TSD left ventricular ejection time predicted later R1 sleep offset, longer sleep duration, and higher WASO; by contrast, higher TSD AM and TSD PM heart rate predicted earlier R1 sleep offset. TSD CV indices explained 14.8%–24.9% of the R1 sleep variance. Notably, females showed significant predictive bidirectional relationships. Our novel results demonstrate that baseline sleep predicts CV metrics during TSD and psychological stress, and that these metrics predict recovery sleep, underscoring crucial relationships, mechanisms, and biomarkers between sleep and cardiovascular health.

## 1 INTRODUCTION

According to the American Heart Association, sleep duration is one of the essential eight elements for evaluating cardiovascular (CV) health (Lloyd‐Jones et al., 2022). Moreover, chronic sleep loss is a critical public health concern related to CV disease (Cappuccio et al., 2011; Liew & Aung, 2021; Liu & Chen, 2019; Mullington et al., 2009; Tobaldini et al., 2017). Past studies have shown short‐term and long‐term trait‐like stability of CV and actigraphic sleep measures across repeated exposures to total sleep deprivation (TSD) and recovery conditions (Pasetes et al., 2023a; Pasetes & Goel, 2024). Prior research has also shown changes in CV indices after one night of TSD (Kato et al., 2000; Kuetting et al., 2019; Mikulski et al., 2006; Pasetes et al., 2023b; Yamazaki, Rosendahl‐Garcia, et al., 2022) and after one night of TSD and psychological stress (Yamazaki, Rosendahl‐Garcia, et al., 2022). Furthermore, both short‐term and long‐term stress affect CV indices and increase the risk for CV disease (Turner et al., 2020). A well‐validated acute psychological stressor, the Trier Social Stress Test (Kirschbaum et al., 1993), increases blood pressure, heart rate (HR), vascular resistance, and cardiac output and decreases left ventricular ejection time (LVET) and stroke volume (SV) (Allen et al., 2014; Jayasinghe et al., 2017). Of note, TSD and various stress conditions combined also increase blood pressure (Bozer et al., 2021; Kato et al., 2000).

Previous studies have examined relationships between sleep and blood pressure and/or HR measures in conditions without sleep loss (Abbott et al., 2019; Bigalke et al., 2024; Chang & Kang, 2021; Doyle et al., 2019; Ekstedt et al., 2004; Fujikawa et al., 2009; Jansen et al., 2020; Liang et al., 2024; Mezick et al., 2012; Sekiguchi et al., 2019; Springall De Pablo & Lauderdale, 2024; Yiallourou et al., 2021), and a few studies have examined these relationships after psychological stress (Eiman et al., 2019; Massar et al., 2017; Mezick et al., 2014). Only one study found an association between self‐rated sleep quality and blood pressure after a night of sleep restriction (Bommarito & Millar, 2024). No studies, however, have examined predictors between baseline sleep and CV metrics including novel echocardiographic measures during acute TSD and psychological stress. Furthermore, it is important to examine these CV predictors during TSD to identify biomarkers and highlight critical relationships between metrics of sleep health and cardiovascular health.

For the first time, we examined whether baseline actigraphic sleep measures the night before TSD acutely predicted blood pressure and echocardiographic CV measures during TSD and psychological stress in healthy adults under controlled conditions. Furthermore, in order to determine if those predictors that were significant were unique to TSD in the morning and/or TSD and psychological stress in the evening, we also evaluated the extent to which baseline actigraphic sleep measures predicted CV indices during the baseline morning before exposure to TSD. In addition, this is the first assessment of whether CV indices during TSD and psychological stress acutely predicted sleep measures during that night's recovery. We hypothesized that (1) better sleep health metrics the night before TSD would significantly predict better CV health metrics during TSD in the morning and during TSD and psychological stress in the evening but not during baseline; and (2) better CV health metrics during TSD in the morning and during TSD and psychological stress in the evening would significantly predict better subsequent recovery sleep health metrics.

## 2 MATERIALS AND METHODS

### 2.1 Participants

The Human Research Program Human Exploration Research Analog (HERA), located in Johnson Space Center in Houston, TX, is a high‐fidelity space analog isolation facility. In this highly controlled facility, we studied 32 healthy adults (ages 27–53; mean age ± standard deviation [SD], 35.1 ± 7.1 years, 14 females: 35.8 ± 7.6 years; 18 males: 34.6 ± 7.0 years). Four participants at a time took part in one of four HERA 14‐day studies or one of four HERA 30‐day studies. Participants were screened extensively by the National Aeronautics and Space Administration (NASA) and had human‐support skills and technical and/or scientific backgrounds relevant for space exploration. Inclusion/exclusion criteria included enrolling both males and females; all participants were in excellent health—they passed a physical exam, a psychological assessment, and a drug screen, and had no history of CV, musculoskeletal, neurological, integumentary, or gastrointestinal problems (Abeln et al., 2022; Pasetes et al., 2023a, 2023b; Saveko et al., 2022; Yamazaki, Antler, Casale, et al., 2021; Yamazaki, Rosendahl‐Garcia, et al., 2022). The study was approved by the Institutional Review Boards of NASA, who had primary oversight, and by the University of Pennsylvania, and all protocol methods were carried out in accordance with approved guidelines and regulations. Prior to inclusion in the study, participants provided written informed consent, which was in accordance with the Declaration of Helsinki. Participants were compensated for their participation in the protocol.

### 2.2 Procedures

**FIGURE 1:**
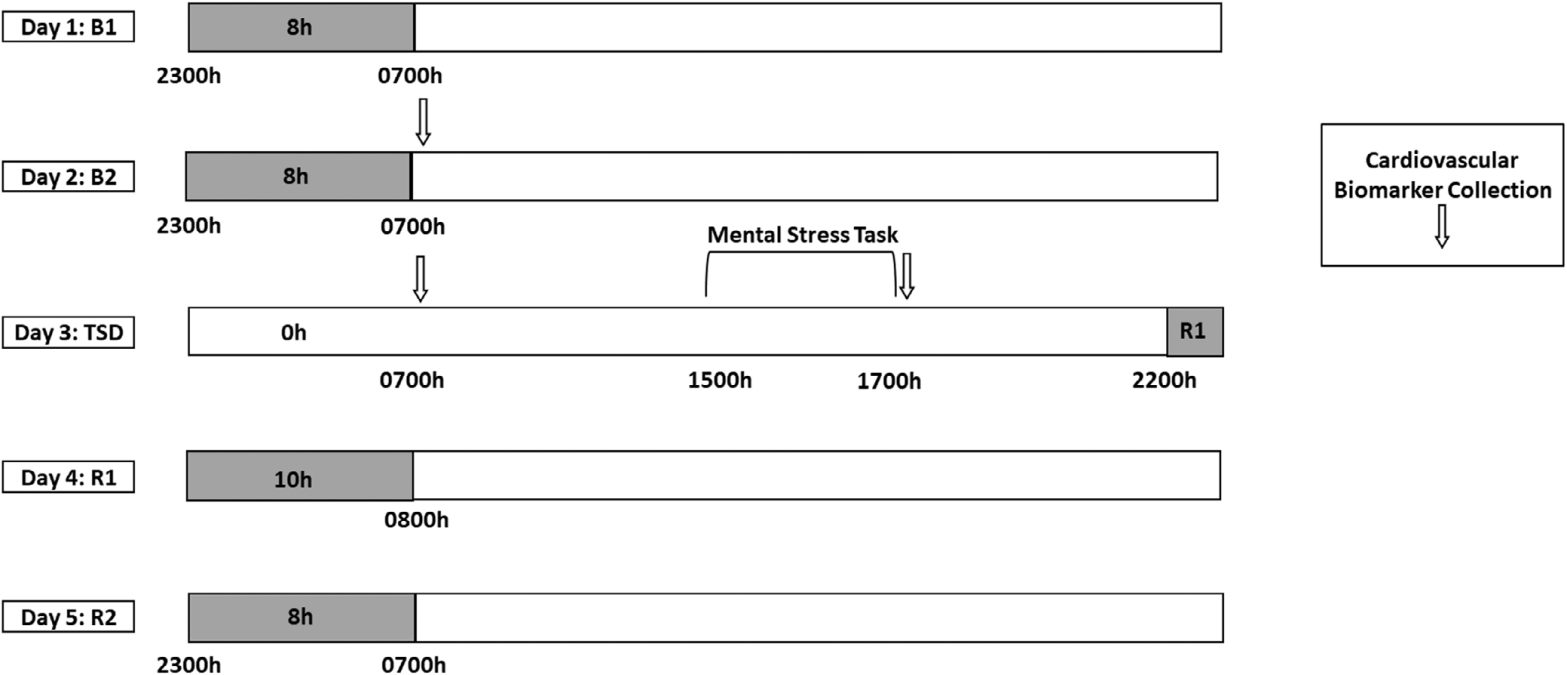
Consecutive 5‐day experimental protocol. Actigraphic nighttime sleep data were collected during the pre‐experimental phase, which occurred 8–10 days prior to the first baseline night of the consecutive 5‐day experiment (not shown in the figure). During the consecutive 5‐day experiment, participants received two nights of baseline with an 8‐h time‐in‐bed sleep opportunity (B1, B2; 2300–0700 h). Baseline cardiovascular (CV) measure collection occurred at approximately 0700 h (white arrow) after the B2 night. Following B2 daytime, participants experienced continued wakefulness for approximately 39 h of total sleep deprivation (TSD). CV morning measure collection occurred at approximately 0700 h after approximately 25 h of TSD (white arrow). A mental stress task (bracket) was administered between 1500 and 1700 h during the TSD day, with CV measure collection after mental stress task completion at approximately 1700 h following approximately 34 h of TSD (white arrow). Recovery sleep opportunities, which followed approximately 39 h of TSD, were 10‐h time‐in‐bed (recovery 1 [R1]; 2200–0800 h) and 8‐h time‐in‐bed (recovery 2 [R2]; 2300–0700 h). Actigraphic sleep data were collected during the nighttime time‐in‐bed opportunities (shaded gray bars).

During each HERA study, participants took part in a 5‐day experiment that induced total sleep deprivation and psychological stress (Figure 1). The 5‐day experiment occurred during study days 9–13 within the 14‐day study and during study days 23–27 within the 30‐day study and consisted of 2 baseline nights [B1 and B2; 8‐h time‐in‐bed, 2300–0700 h], followed by 39‐h acute TSD during which participants remained awake. A mental stress task was conducted between approximately 1500–1700 h on the day after the TSD night to produce psychological stress (described below). TSD was followed by a 10‐h time‐in‐bed recovery night (R1; 2200–0800 h), and a second 8‐h time‐in‐bed recovery night (R2; 2300–0700 h). Of note, due to the homeostatic pressure to sleep following TSD, participants were allowed flexibility in bedtimes and waketimes on the R1 night (Table 1). Fitness levels were not specifically measured; however, all participants experienced similar amounts of activity during the study, were confined to engaging in prescribed activities at specific times, and were prohibited from napping or consuming caffeine during the experiment. Participants were monitored continuously by wrist actigraphy and by outside observers to ensure adherence.

**TABLE 1:** Mean ± SD actigraphic sleep measures during the pre‐experimental phase, baseline night 1 (B1), baseline night 2 (B2), and recovery night 1 (R1).

Sleep measure	Pre‐experimental phase	B1	B2	TSD	R1
Sleep duration (min)^c^	430.515 ± 19.083	444.760 ± 28.961^b^	443.258 ± 31.620	‐	590.660 ± 41.732^b^
Sleep onset (clock hour)^c^	23.607 ± 0.341	23.398 ± 0.388^a^	23.415 ± 0.534	‐	22.155 ± 0.410^b^
Sleep offset (clock hour)	6.783 ± 0.133	6.820 ± 0.249^a^	6.803 ± 0.214	‐	7.999 ± 0.561^b^
Sleep midpoint (clock hour)	3.195 ± 0.204	3.112 ± 0.221^a^	3.118 ± 0.311	‐	3.096 ± 0.320^b^
Sleep efficiency (%)	87.552 ± 4.224	88.160 ± 6.271^a^	88.557 ± 4.711^a^	‐	90.840 ± 6.249^a^
WASO (min)	39.302 ± 17.460	37.430 ± 20.520^a^	38.161 ± 20.062	‐	47.200 ± 42.128^a^
Sleep onset latency (min)	8.766 ± 7.419	11.290 ± 18.199^a^	7.678 ± 8.444^a^	‐	1.870 ± 3.401^a^
Percent sleep (%)	90.877 ± 3.957	91.640 ± 4.468^a^	91.424 ± 4.248	‐	92.130 ± 6.049^a^

*Note*: The pre‐experimental phase was the average across 8–10 days before the start of the 5‐day experiment. *N* = 31.

Abbreviations: B1, baseline night 1; B2, baseline night 2; R1, recovery night 1; TSD, total sleep deprivation; WASO, wake after sleep onset.

^a^ *N* = 30.

^b^ *N* = 29.

^c^ Sleep duration [*F*_(2,56)_ = 5.135, *p* = 0.009] and sleep onset [*F*_(2,58)_ = 5.760, *p* = 0.005] demonstrated a significant effect across the pre‐experimental phase, B1, and B2 assessed by repeated measures ANOVA. The pre‐experimental phase sleep duration was significantly shorter than B2 sleep duration as assessed by post hoc analyses with Bonferroni corrections (*p* = 0.021). The pre‐experimental phase sleep onset was significantly later than B1 (*p* = 0.050) and B2 (*p* = 0.024) sleep onset as assessed by post hoc analyses with Bonferroni corrections.

### 2.3 Actigraphy sleep measure collections

A wrist accelerometer (Actiwatch Spectrum, Philips Respironics Healthcare, Bend, OR, USA) measured sleep–wake metrics related to quantity, timing, and quality including sleep duration (the amount of time elapsed between sleep onset and sleep offset), sleep onset (clock time at the start of each sleep period), sleep offset (clock time at the end of each sleep period), sleep midpoint (clock time of the half‐way point between sleep onset and sleep offset; a proxy of circadian phase), sleep efficiency (the percentage of time spent asleep out of the total sleep time), wake after sleep onset (WASO; amount of time awake after initially falling asleep), sleep onset latency (the amount of time it takes to fall asleep), and percent sleep (the percentage of time spent asleep during the sleep period) during the pre‐experimental phase (the average across 8–10 days before the start of the 5‐day experiment), and the B1, B2, and R1 nights (Pasetes et al., 2023a, 2023b; Pasetes & Goel, 2024). Notably, the pre‐experimental phase, and B1 and B2 nights had comparable actigraphic sleep data (Table 1). Actiwatches were worn on the non‐dominant wrist and data were collected in 1‐min intervals (using firmware version 01.01.0015, medium wake threshold) and processed using the Actiware software (version 6.1.0). Actigraphic sleep data during the nighttime intervals were analyzed similar to our prior research (e.g., Dennis et al., 2017; Moreno‐Villanueva et al., 2018; Pasetes et al., 2023a, 2023b; Pasetes & Goel, 2024; Yamazaki, Antler, Lasek, et al., 2021; Yamazaki, Casale, et al., 2022; Yamazaki & Goel, 2020; Yamazaki, Rosendahl‐Garcia, et al., 2022). Of note, actigraphic data from past studies with a similar experimental design using the same Actiwatch had sleep measures within similar ranges (Pasetes et al., 2023a, 2023b; Pasetes & Goel, 2024).

### 2.4 Cardiovascular measure collections

During these experiments, CV indices including systemic vascular resistance (SVRI), SV, LVET, HR, and cardiac index were collected via echocardiography. Systolic and diastolic blood pressure and mean arterial pressure were collected via blood pressure monitor. CV measures were collected in a seated position during the B2 morning (B2 AM), TSD morning (TSD AM; after approximately 25 h of TSD) and TSD evening after a mental stress task (TSD PM; after approximately 34 h of TSD and psychological stress). The B2 AM and TSD AM collections were completed at approximately 0700 h before eating, and the TSD PM collection was completed at approximately 1700 h under highly controlled conditions (Figure 1). All participants fasted for approximately 10 h prior to the AM collections and for approximately 5 h prior to the PM collection to maintain consistency across the study and among participants. Exercise did not occur before the B2 AM, TSD AM, or TSD PM CV measure collections. Of note, all CV measures were within the ranges reported for healthy adults (Table 2) (Cattermole et al., 2017; Klabunde, 2012; Lü et al., 2018; Pasetes et al., 2023a, 2023b; Shaffer & Ginsberg, 2017; Yamazaki, Rosendahl‐Garcia, et al., 2022).

**TABLE 2:** Mean ± SD cardiovascular measures during total sleep deprivation morning (TSD AM) and total sleep deprivation and psychological stress (TSD PM).

Cardiovascular measure	TSD AM	TSD PM
Systemic vascular resistance index (mmHg·L/min/m^2^)	40.201 ± 14.697	38.186 ± 10.768
Stroke volume (mL)	68.009 ± 15.848	65.309 ± 15.113
Left ventricular ejection time (msec)	329.330 ± 35.304	313.806 ± 29.362
Heart rate (beats/min)	65.167 ± 10.206	70.272 ± 11.058
Cardiac index (L/min/m^2^)	2.418 ± 0.643	2.501 ± 0.697
Systolic blood pressure (mmHg)	114.767 ± 10.950^a^	114.533 ± 8.939^a^
Diastolic blood pressure (mmHg)	77.733 ± 7.428^a^	77.300 ± 6.374^a^
Mean arterial blood pressure (mmHg)	90.067 ± 8.077^a^	89.800 ± 6.609^a^

*Note*: *N* = 31.

Abbreviation: TSD, total sleep deprivation.

^a^ *N* = 30.

### 2.5 Echocardiogram procedures

Due to strict isolation conditions in the facility, one participant collected all cardiac ultrasound images on the other participants during each study, and a second participant collected all cardiac ultrasound images on the primary collector during each study. All ultrasound operators were extensively trained to obtain ultrasound images and Doppler prior to the study and repeated collection procedures uniformly across each time point (Pasetes et al., 2023a, 2023b; Yamazaki, Rosendahl‐Garcia, et al., 2022). Notably, we have demonstrated robust consistency of CV measures across time based on this collection method in our prior research (Pasetes et al., 2023a).

SV was collected via ultrasound imaging (GE Vivid q ultrasound system [General Electric Medical Systems, Milwaukee]) in a seated posture across all time points (Arbeille & Herault, 1998; Ihlen et al., 1984; McLennan et al., 1986; Yamazaki, Rosendahl‐Garcia, et al., 2022). Two‐dimensional images of the left ventricular outflow tract were collected from each participant using a 5S‐RS transducer (Pasetes et al., 2023a, 2023b; Yamazaki, Rosendahl‐Garcia, et al., 2022). The left ventricular outflow tract was imaged from the parasternal long‐axis view while the participants were semi‐supine in a left lateral decubitus posture (Pasetes et al., 2023a, 2023b; Yamazaki, Rosendahl‐Garcia, et al., 2022). Three to four, two‐second cine‐loops of dynamic motion of the left ventricular outflow tract were digitally saved. SV was collected utilizing a continuous wave pencil (Pedof) probe for Doppler interrogation (Pasetes et al., 2023a, 2023b; Yamazaki, Rosendahl‐Garcia, et al., 2022). Continuous wave Doppler signals were taken from the ascending aorta at the suprasternal notch in a seated posture (Pasetes et al., 2023a, 2023b; Yamazaki, Rosendahl‐Garcia, et al., 2022). Three five‐second cine‐loop sweeps of continuous wave Doppler data were collected and digitally stored as proprietary raw data (Pasetes et al., 2023a, 2023b; Yamazaki, Rosendahl‐Garcia, et al., 2022).

A professional sonographer (K.M.R‐G) conducted formal analyses of the echocardiography data. Analysis of the digital data was performed using Echo PAC PC (BT12) software (General Electric Medical Systems, Milwaukee, WI, United States). Left ventricular outflow tract diameters were measured just proximal to the aortic valve leaflet insertion from three consecutive cine‐loops at the maximum opening of the aortic valve. Five consecutive continuous wave Doppler waveform profiles were traced to calculate the velocity time integral. The interval between each maximum peak on the Doppler spectral from the ascending aorta was used to calculate HR. The duration of each beat was measured to determine LVET for each SV. The velocity time integral and LVET were then transferred from the Echo PAC software to Excel to calculate SV and cardiac index using the following formulas:

$$\begin{array}{c} \mathrm{SV}=\left(\text{left ventricular outflow tract cross sectional area}\right)\\ \times \;\text{velocity time integral }\\ \text{cardiac index}=\left(\left(\mathrm{SV}\times \mathrm{HR}\right)/1000\right)/\text{body surface area} \end{array}$$

Any further continuous wave Doppler waveforms not included in the consecutive SV analysis were analyzed for HR in a seated posture where available.

### 2.6 Blood pressure and systemic vascular resistance index

Brachial systolic blood pressure and diastolic blood pressure were recorded using an Omron BP791IT 10 series Plus Automatic Blood Pressure Monitor with ComFit™ Cuff (Lake Forest, IL, United States) in a seated position on the non‐dominant arm (Pasetes et al., 2023a, 2023b; Yamazaki, Rosendahl‐Garcia, et al., 2022). Participants were seated for 3 min before blood pressure collection. The average value of three consecutive readings, taken 1 min apart, was used for analyses. SVRI was calculated by assuming that central venous pressure was zero and by using the following equation, whereby mean arterial pressure = (systolic blood pressure + 2 × diastolic blood pressure)/3 (Klabunde, 2012; Norsk et al., 2015; Pasetes et al., 2023a, 2023b; Yamazaki, Rosendahl‐Garcia, et al., 2022):

SVRI=mean arterial pressure/cardiac index

### 2.7 Mental stress task

The mental stress task implemented was similar to the Trier Social Stress Test, a commonly used and validated test to experimentally induce psychological stress (Allen et al., 2014; Kirschbaum et al., 1993; Yamazaki, Antler, Casale, et al., 2021; Yamazaki, Rosendahl‐Garcia, et al., 2022), which has been successfully validated and modified using a virtual, rather than a physical audience (Helminen et al., 2021; Kelly et al., 2007; Ruiz et al., 2010; Yamazaki, Antler, Casale, et al., 2021; Yamazaki, Rosendahl‐Garcia, et al., 2022). Participants individually received the 30‐min mental stress task. Participants were given time to prepare their thoughts about how they responded to and felt about the TSD, and then they were interviewed by the research team, including one of the authors (N.G.). The interview involved numerous difficult questions regarding responses to TSD, including those related to motivation, performance, aptitude, and interactions with others. These questions were followed by multiple challenging cognitive tests, which included a 5‐min calculation task involving counting backward aloud in 13‐step sequences and a 3‐min Stroop task; all these tests were added to induce high cognitive performance stress and workload. The mental stress task was conducted with participants remotely via audio and a one‐way video camera due to the isolation conditions (Moreno‐Villanueva et al., 2018; Yamazaki, Antler, Casale, et al., 2021; Yamazaki, Rosendahl‐Garcia, et al., 2022).

### 2.8 Statistical analyses

All statistical analyses were performed using SPSS v29 (SPSS Inc., Chicago, IL, USA), with *p* < 0.05 considered statistically significant. Prior studies have found normal distributions for the CV and sleep measures examined in these studies (Orme et al., 1999; Pasetes et al., 2023a, 2023b; Pasetes & Goel, 2024; Yamazaki, Rosendahl‐Garcia, et al., 2022).

For this paper, we analyzed the bidirectional predictors and relationships between B2 and R1 actigraphic night sleep measures and CV measures during TSD (TSD AM) and TSD and psychological stress (TSD PM). Pearson's correlation coefficient (*r*) and Pearson's *R*‐squared (*R* ^2^; coefficient of determination) assessed relationships between B2 actigraphic night sleep metrics (the last sleep opportunity prior to TSD) and TSD AM and TSD PM CV measures and between TSD AM and TSD PM CV measures and R1 actigraphic night sleep indices. For significant associations determined via Pearson's correlations, simple linear regression analyses were conducted (Fonseca et al., 2024). In the results section and in the tables, we present the unstandardized beta (*β*), which represents the regression slope, and which was used to interpret our findings. The standardized beta represents the “unit‐free” measure of effect size, which is equivalent to Pearson's correlation.

Simple linear regression analyses evaluated the extent to which each specific B2 actigraphic night sleep measure (independent variable) predicted each CV measure during TSD AM and TSD PM (dependent variable) and evaluated the extent to which each CV measure during TSD AM and TSD PM (independent variable) predicted each R1 actigraphic night sleep measure (dependent variable). In addition, given the well‐established sex differences in objective sleep measures (Forshaw et al., 2024; Johnson et al., 2024; Lok et al., 2024; Mulè et al., 2021; Roberts et al., 2022; Wright et al., 2023) and in blood pressure and echocardiographic CV measures (Drury et al., 2024; Hoopes et al., 2021; Liang et al., 2024; Lin et al., 2023; Quer et al., 2020; Wooten et al., 2021), we conducted exploratory simple linear regression analyses on the significant main simple linear regression analyses in males and females separately to determine if these predictors remained significant. Furthermore, in order to determine if the significant results were exclusive to TSD AM and/or TSD PM CV measures, simple linear regression analyses evaluated whether B2 actigraphic sleep measures predicted CV indices during the B2 morning (B2 AM) before exposure to TSD.

Pearson's correlation coefficient (*r*) also assessed relationships between B2 night sleep metrics and between R1 night sleep metrics. Repeated Measures (RM) ANOVA assessed potential differences in sleep metrics across the pre‐experimental phase and the B1 and B2 nights (Table 1). Sphericity Assumed corrections for degrees of freedom were applied for all RM ANOVAs since Mauchly's test was never violated for significant within‐subject effects. Post hoc analyses with Bonferroni corrections compared the pre‐experimental phase and the B1 and B2 nights when there was a significant effect across all three phases. Bonferroni‐corrected *p* values are reported.

For *N* = 1 participant, B2 sleep onset latency and B2 sleep efficiency measures were outliers (±3 SD from the mean) and therefore were excluded from all B2 analyses. For *N* = 1 participant, R1 sleep duration, R1 sleep offset, and R1 sleep midpoint were outliers (±3 SD from the mean) and therefore were excluded from all R1 analyses (in addition, since sleep offset and sleep midpoint were both outliers, sleep onset was excluded). *N* = 1 participant was removed from all B2 and R1 analyses due to four actigraphic sleep measure outliers (±3 SD from the mean) and *N* = 1 participant did not wear the Actiwatch during R1 and was therefore excluded from all R1 analyses. *N* = 1 participant was removed from TSD AM and TSD PM systolic blood pressure, diastolic blood pressure, and mean arterial pressure analyses due to outliers (±3 SD from the mean).

## 3 RESULTS

### 3.1 B2 actigraphic night sleep measures as predictors of cardiovascular indices during TSD and TSD and psychological stress

**FIGURE 2:**
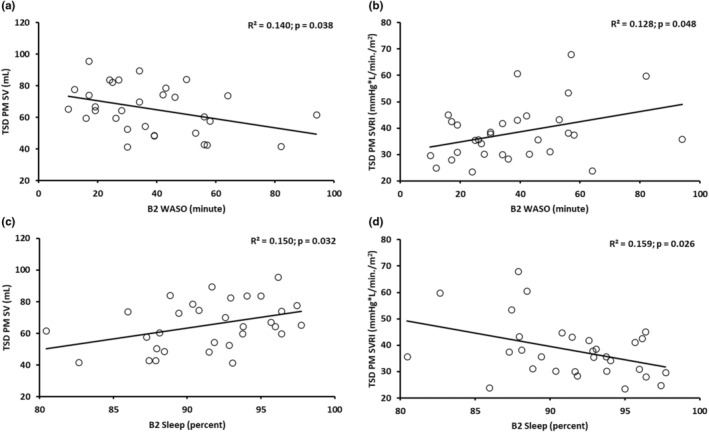
Scatter plots displaying baseline night 2 (B2) actigraphic sleep measures as significant predictors of cardiovascular (CV) measures derived from echocardiography after 34 h of total sleep deprivation and psychological stress (TSD PM). Simple linear regression found that higher B2 wake after sleep onset (WASO) significantly predicted (a) lower TSD PM stroke volume (SV) and (b) higher TSD PM systemic vascular resistance index (SVRI). Greater B2 percent sleep significantly predicted (c) higher TSD PM SV and (d) lower TSD PM SVRI. The regression line, *R* ^2^ and *p* value for each plot are shown. *p* < 0.05 was significant. *N* = 31 for a–d.

Simple linear regression analyses evaluated the extent to which B2 actigraphic night sleep measures predicted CV indices during TSD in the morning (TSD AM) and during TSD and psychological stress in the evening (TSD PM). Higher B2 WASO significantly predicted lower TSD PM SV (*r* = −0.374) and higher TSD PM SVRI (*r* = 0.358): each 1‐min increase in B2 WASO significantly predicted a 0.281 mL decrease in TSD PM SV and a 0.192 mmHg·L/min/m^2^ increase in TSD PM SVRI (Table 3 and Figure 2a,b). B2 WASO predicted 14.0% of the variance in TSD PM SV and 12.8% of the variance in TSD PM SVRI. In contrast to the WASO findings, greater B2 percent sleep significantly predicted higher SV (*r* = 0.387) and lower SVRI (*r* = −0.398) during TSD PM: each 1% increase in B2 percent sleep significantly predicted a 1.376 mL increase in TSD PM SV and a 1.010 mmHg·L/min/m^2^ decrease in TSD PM SVRI (Table 3 and Figure 2c,d). B2 percent sleep predicted 15.0% of the variance in TSD PM SV and 15.9% of the variance in TSD PM SVRI. We also found in females each 1‐min increase in B2 WASO significantly predicted a 0.478 mmHg·L/min/m^2^ increase in TSD PM SVRI (*r* = 0.659; *R* ^2^ = 0.435; Table 4) and each 1% increase in B2 percent sleep significantly predicted a 2.213 mmHg·L/min/m^2^ decrease in TSD PM SVRI (*r* = −0.652; *R* ^2^ = 0.425; Table 4).

**TABLE 3:** Baseline night 2 (B2) actigraphic sleep metrics as significant predictors of total sleep deprivation and psychological stress (TSD PM) cardiovascular (CV) measures.

B2 sleep predictor and TSD CV measure (*N* = 31)	Unstandardized *β* ± SE	Standardized beta	*t*	95% CI	*p* (two‐tailed)
B2 WASO and TSD PM SV	−0.281 ± 0.130	−0.374	−2.169	−0.547, −0.016	**0.038**
B2 WASO and TSD PM SVRI	0.192 ± 0.093	0.358	2.065	0.002, 0.382	**0.048**
B2 % Sleep and TSD PM SV	1.376 ± 0.609	0.387	2.258	0.130, 2.622	**0.032**
B2 % Sleep and TSD PM SVRI	−1.010 ± 0.432	−0.398	−2.340	−1.893, −0.127	**0.026**

*Note*: Boldface indicates significant *p* values at *p* < 0.05.

Abbreviations: B2, baseline night 2; CI, confidence interval; CV, cardiovascular; SE, standard error; SV, stroke volume; SVRI, systemic vascular resistance index; TSD, total sleep deprivation; WASO, wake after sleep onset.

**TABLE 4:** Exploratory sex differences in baseline night 2 (B2) actigraphic sleep metrics as significant predictors of total sleep deprivation and psychological stress (TSD PM) cardiovascular (CV) measures.

B2 sleep predictor and TSD CV measure	Unstandardized *β* ± SE	Standardized beta	*t*	95% CI	*p* (two‐tailed)
B2 WASO and TSD PM SV					
Females	−0.443 ± 0.235	−0.495	−1.888	−0.960, 0.073	0.086
Males	−0.246 ± 0.165	−0.349	−1.489	−0.596, 0.104	0.156
B2 WASO and TSD PM SVRI					
Females	0.478 ± 0.164	0.659	2.909	0.116, 0.840	**0.014**
Males	0.090 ± 0.112	0.196	0.799	−0.148, 0.327	0.436
B2 % Sleep and TSD PM SV					
Females	1.955 ± 1.119	0.466	1.747	−0.508, 4.417	0.108
Males	1.265 ± 0.773	0.379	1.637	−0.373, 2.903	0.121
B2 % Sleep and TSD PM SVRI					
Females	−2.213 ± 0.777	−0.652	−2.850	−3.922, −0.504	**0.016**
Males	−0.568 ± 0.522	−0.262	−1.087	−1.674, 0.539	0.293

*Note*: Boldface indicates significant *p* values at *p* < 0.05. Females, *N* = 13; Males, *N* = 18.

Abbreviations: B2, baseline night 2; CI, confidence interval; CV, cardiovascular; SE, standard error; SV, stroke volume; SVRI, systemic vascular resistance index; TSD, total sleep deprivation; WASO, wake after sleep onset.

There were no other significant B2 actigraphic night sleep measures as predictors of CV indices during TSD and TSD and psychological stress, and no other significant predictors in females and males.

### 3.2 B2 actigraphic night sleep measures as predictors of B2 morning cardiovascular indices

In order to determine if the aforementioned significant predictors were unique to TSD AM and/or TSD PM CV indices, simple linear regression analyses evaluated the extent to which B2 actigraphic night sleep measures predicted CV indices during the B2 morning (B2 AM) before exposure to TSD. We found one significant result in which higher B2 WASO significantly predicted higher B2 AM SVRI: each 1‐min increase in B2 WASO significantly predicted a 0.309 mmHg·L/min/m^2^ increase in B2 AM SVRI (*r* = 0.414; *R* ^2^ = 0.171; β ± SE = 0.309 ± 0.126; *t* = 2.450; *p* = 0.021). There were no other significant B2 actigraphic night sleep measures as predictors of B2 AM CV indices.

### 3.3 Cardiovascular indices during TSD and TSD and psychological stress as predictors of R1 actigraphic sleep measures

**FIGURE 3:**
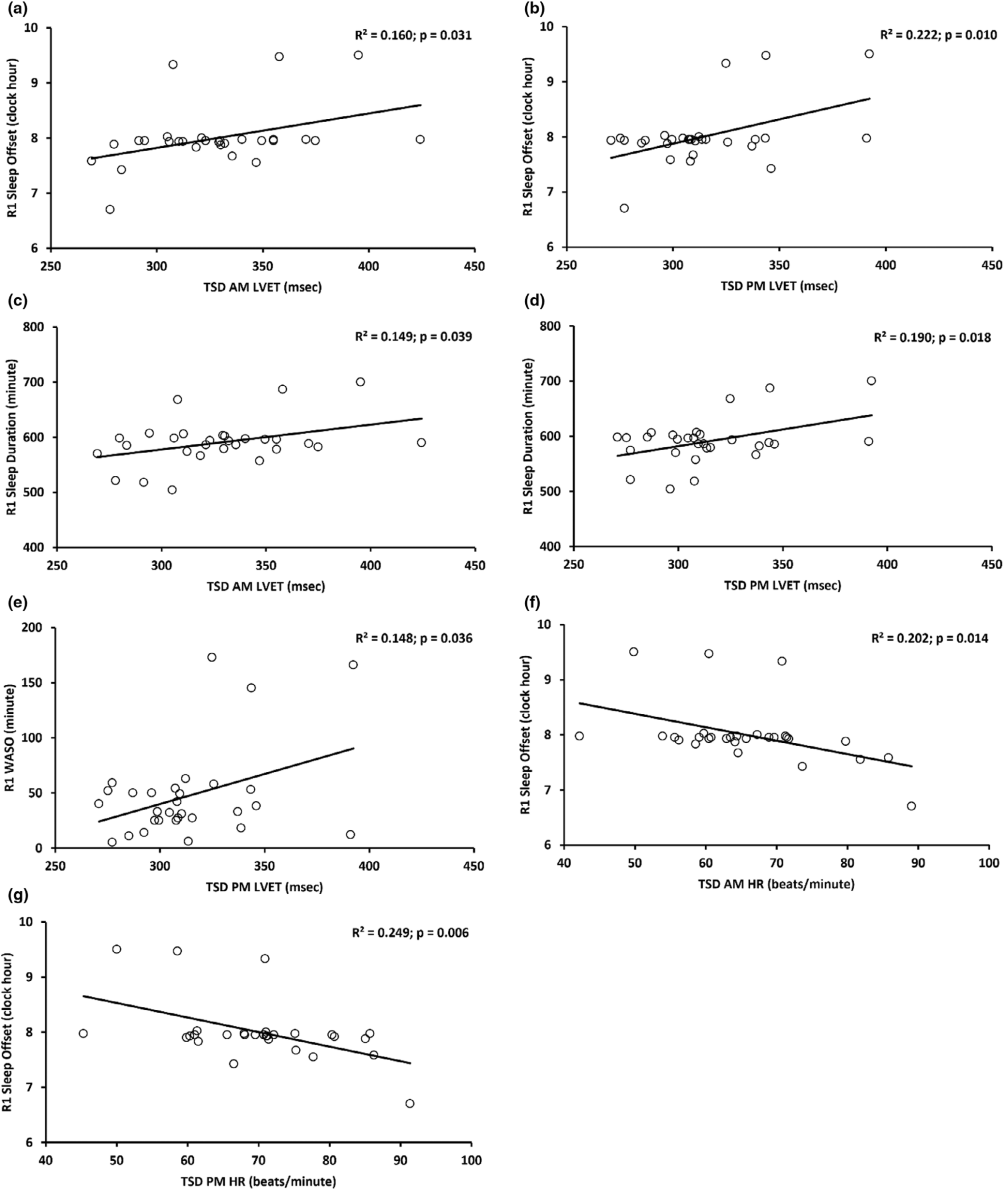
Scatter plots displaying cardiovascular (CV) measures derived from echocardiography after 25 h of total sleep deprivation (TSD AM) and after 34 h of TSD and psychological stress (TSD PM) as significant predictors of recovery night 1 (R1) actigraphic sleep measures. Simple linear regression found that longer TSD AM and TSD PM left ventricular ejection time (LVET) predicted (a, b) later R1 sleep offset and (c, d) longer R1 sleep duration; and longer TSD PM LVET predicted (e) higher R1 wake after sleep onset (WASO). Higher TSD AM and TSD PM heart rate (HR) predicted (f, g) earlier R1 sleep offset. The regression line, *R* ^2^ and *p* value for each plot are shown. *p* < 0.05 was significant. *N* = 29 for a–d, f, g. *N* = 30 for e.

Simple linear regression analyses evaluated the extent to which CV indices during TSD in the morning (TSD AM) and TSD and psychological stress in the evening (TSD PM) predicted R1 actigraphic night sleep measures. Longer TSD AM and TSD PM LVET significantly predicted later R1 sleep offset and longer R1 sleep duration (*r*: 0.386–0.471): each 1 msec increase in TSD AM and TSD PM LVET predicted a 0.006–0.009 h (0.36–0.54 min) delay in R1 sleep offset, respectively (Table 5 and Figure 3a,b) and a 0.449–0.607 min increase in R1 sleep duration, respectively (Table 5 and Figure 3c,d). TSD AM and TSD PM LVET predicted 16.0%–22.2% of the variance in R1 sleep offset and 14.9%–19.0% of the variance in R1 sleep duration. Moreover, in females, each 1 msec increase in TSD AM and TSD PM LVET significantly predicted a 0.013–0.015 h (0.78–0.90 min) delay in R1 sleep offset (*r* = 0.638; *R* ^2^ = 0.407), respectively (*r* = 0.626; *R* ^2^ = 0.392; Table 6) and a 0.941 min (*r* = 0.609; *R* ^2^ = 0.371) to 1.075 min increase in R1 sleep duration, respectively (*r* = 0.602; *R* ^2^ = 0.362; Table 6).

**TABLE 5:** Cardiovascular (CV) measures during total sleep deprivation (TSD AM) and TSD and psychological stress (TSD PM) as significant predictors of recovery night 1 (R1) actigraphic sleep metrics.

TSD CV predictor and R1 sleep measure (*N* = 30)	Unstandardized *β* ± SE	Standardized beta	*t*	95% CI	*p* (two‐tailed)
TSD AM LVET and R1 sleep offset^a^	0.006 ± 0.003	0.401	2.272	0.001, 0.012	**0.031**
TSD PM LVET and R1 sleep offset^a^	0.009 ± 0.003	0.471	2.776	0.002, 0.015	**0.010**
TSD AM LVET and R1 sleep duration^a^	0.449 ± 0.207	0.386	2.173	0.025, 0.872	**0.039**
TSD PM LVET and R1 sleep duration^a^	0.607 ± 0.241	0.436	2.517	0.112, 1.101	**0.018**
TSD PM LVET and R1 WASO	0.545 ± 0.247	0.385	2.207	0.039, 1.051	**0.036**
TSD AM HR and R1 sleep offset^a^	−0.024 ± 0.009	−0.449	−2.614	−0.044, −0.005	**0.014**
TSD PM HR and R1 sleep offset^a^	−0.026 ± 0.009	−0.499	−2.992	−0.045, −0.008	**0.006**

*Note*: Boldface indicates significant *p* values at *p* < 0.05.

Abbreviations: CI, confidence interval; CV, cardiovascular; HR, heart rate; LVET, left ventricular ejection time; R1, recovery night 1; SE, standard error; TSD, total sleep deprivation; WASO, wake after sleep onset.

^a^ *N* = 29.

**TABLE 6:** Exploratory sex differences in cardiovascular (CV) measures during total sleep deprivation (TSD AM) and TSD and psychological stress (TSD PM) as significant predictors of recovery night 1 (R1) actigraphic sleep metrics.

TSD CV predictor and R1 sleep measure	Unstandardized *β* ± SE	Standardized beta	*t*	95% CI	*p* (two‐tailed)
TSD AM LVET and R1 sleep offset					
Females	0.013 ± 0.005	0.638	2.620	0.002, 0.024	**0.026**
Males^a^	0.002 ± 0.003	0.198	0.781	−0.004, 0.009	0.447
TSD PM LVET and R1 sleep offset					
Females	0.015 ± 0.006	0.626	2.538	0.002, 0.027	**0.029**
Males^a^	0.005 ± 0.004	0.295	1.197	−0.004, 0.013	0.250
TSD AM LVET and R1 sleep duration					
Females	0.941 ± 0.388	0.609	2.427	0.077, 1.804	**0.036**
Males^a^	0.176 ± 0.230	0.193	0.762	−0.315, 0.666	0.458
TSD PM LVET and R1 sleep duration					
Females	1.075 ± 0.451	0.602	2.385	0.071, 2.079	**0.038**
Males^a^	0.300 ± 0.294	0.255	1.021	−0.326, 0.926	0.324
TSD PM LVET and R1 WASO					
Females	1.182 ± 0.410	0.673	2.881	0.268, 2.097	**0.016**
Males	0.140 ± 0.318	0.110	0.441	−0.533, 0.813	0.665
TSD AM HR and R1 sleep offset					
Females	−0.053 ± 0.019	−0.674	−2.886	−0.095, −0.012	**0.016**
Males^a^	−0.014 ± 0.010	−0.339	−1.396	−0.035, 0.007	0.183
TSD PM HR and R1 sleep offset					
Females	−0.059 ± 0.017	−0.735	−3.429	−0.097, −0.021	**0.006**
Males^a^	−0.015 ± 0.010	−0.362	−1.502	−0.035, 0.006	0.154

*Note*: Boldface indicates significant *p* values at *p* < 0.05. Females, *N* = 12; Males, *N* = 18.

Abbreviations: CI, confidence interval; CV, cardiovascular; HR, heart rate; LVET, left ventricular ejection time; R1, recovery night 1; SE, standard error; TSD, total sleep deprivation; WASO, wake after sleep onset.

^a^ *N* = 17.

In addition, longer LVET during TSD PM significantly predicted higher R1 WASO (r = 0.385): each 1 msec increase in TSD PM LVET significantly predicted a 0.545 min increase in R1 WASO (Table 5 and Figure 3e). TSD PM LVET predicted 14.8% of the variance in R1 WASO. In addition, in females, each 1 msec increase in TSD PM LVET significantly predicted a 1.182 min increase in R1 WASO (*r* = 0.673; *R* ^2^ = 0.454; Table 6).

In contrast to the LVET findings, higher HR during TSD AM (*r* = −0.449) and TSD PM (*r* = −0.499) significantly predicted an earlier R1 sleep offset: each 1 beat/min increase in TSD AM and TSD PM HR significantly predicted a 0.024 h to 0.026 h (1.44–1.56 min) advance in R1 sleep offset, respectively (Table 5 and Figure 3f,g). TSD AM and TSD PM HR predicted 20.2%–24.9% of the variance in R1 sleep offset. We also found in females, each 1 msec increase in TSD AM and TSD PM HR significantly predicted a 0.053 h (3.18 min; *r* = −0.674; *R* ^2^ = 0.454) to 0.059 h (3.54 min; *r* = −0.735; *R* ^2^ = 0.540) advance in R1 sleep offset, respectively (Table 6).

There were no other significant CV indices during TSD and TSD and psychological stress as predictors of R1 actigraphic night sleep measures, and no other significant predictors in females and males.

### 3.4 Correlations between B2 actigraphic night sleep metrics and between R1 actigraphic night sleep metrics

Pearson's correlation analyses found significant correlations between B2 actigraphic night sleep measures (Table 7) and between R1 actigraphic night sleep measures (Table 8).

**TABLE 7:** Pearson's correlation coefficients for baseline night 2 (B2) actigraphic sleep measures.

Sleep measure	Sleep duration	Sleep onset	Sleep offset	Sleep midpoint	Sleep efficiency^c^	Wake after sleep onset	Sleep onset latency^c^
Sleep onset	−0.919^a^						
Sleep offset	0.166	0.237					
Sleep midpoint	−0.696^a^	0.917^a^	0.577^a^				
Sleep efficiency^c^	0.031	0.014	0.091	−0.014			
Wake after sleep onset	0.255	−0.176	0.186	−0.008	−0.848^a^		
Sleep onset latency^c^	−0.338	0.371^b^	0.035	0.282	−0.257	−0.133	
Percent sleep	−0.109	0.038	−0.172	−0.100	0.881^a^	−0.988^a^	0.096

*Note*: *N* = 31.

^a^ *p* < 0.01.

^b^ *p* < 0.05.

^c^ *N* = 30.

**TABLE 8:** Pearson's correlation coefficients for recovery night 1 (R1) actigraphic sleep measures.

Sleep measure	Sleep duration^c^	Sleep onset^c^	Sleep offset^c^	Sleep midpoint^c^	Sleep efficiency	Wake after sleep onset	Sleep onset latency
Sleep onset^c^	−0.592^a^						
Sleep offset^c^	0.809^a^	−0.005					
Sleep midpoint^c^	0.225	0.616^a^	0.729^a^				
Sleep efficiency	−0.525^a^	0.087	−0.588^a^	−0.512^a^			
Wake after sleep onset	0.688^a^	−0.122	0.764^a^	0.608^a^	−0.945^a^		
Sleep onset latency	−0.175	0.455^b^	0.117	0.439^b^	−0.297	0.142	
Percent sleep	−0.613^a^	0.076	−0.705^a^	−0.604^a^	0.973^a^	−0.989^a^	−0.215

*Note*: *N* = 30.

^a^ *p* < 0.01.

^b^ *p* < 0.05.

^c^ *N* = 29.

## 4 DISCUSSION

We demonstrated novel bidirectional predictors and mechanisms between baseline and recovery sleep measures and CV measures during TSD and psychological stress. We found that higher B2 WASO predicted lower TSD PM SV and higher TSD PM SVRI, with greater B2 percent sleep predicting inverse relationships. Overall, B2 sleep measures predicted 12.8–15.9% of the variance in TSD PM CV indices. Of note, we found one significant B2 sleep predictor of baseline CV metrics, whereby higher B2 WASO predicted higher B2 AM SVRI. In addition, longer TSD LVET predicted later R1 sleep offset, longer sleep duration, and higher WASO; by contrast, higher TSD AM and TSD PM HR predicted earlier R1 sleep offset. Overall, TSD CV indices predicted 14.8%–24.9% of the variance in R1 sleep measures. Notably, females showed significant predictive bidirectional relationships . In summary, we demonstrate that baseline sleep measures predict CV metrics during TSD and psychological stress, and that these CV metrics predict subsequent recovery sleep measures. Our results highlight critical mechanisms and biomarkers between sleep health and cardiovascular health.

Our results showed that higher B2 WASO predicted lower TSD PM SV and higher TSD PM SVRI, while greater B2 percent sleep predicted higher TSD PM SV and lower TSD PM SVRI. Consistent with these results, we previously found that SV and SVRI showed opposite changes during TSD in a separate dataset with a similar experimental design (Pasetes et al., 2023b), and furthermore, that TSD AM and TSD PM SV, and TSD AM and TSD PM SVRI did not show significant differences (Yamazaki, Rosendahl‐Garcia, et al., 2022). Notably, our results demonstrate that B2 sleep predicted SV and SVRI during the TSD evening, after exposure to the mental stress task, but not during the morning of TSD. Our TSD SV results may be due to a myocardial mechanism that is invoked in response to stressors that require active coping (James et al., 2012; Lü et al., 2018), such as TSD and psychological stress. Indeed, in an earlier study, we showed that cognitively vulnerable individuals had lower SV and higher SVRI during TSD (Yamazaki, Rosendahl‐Garcia, et al., 2022), thus suggesting these CV responses are adverse rather than adaptive. Importantly, our findings demonstrate that higher WASO and lower percent sleep the night before sleep loss can uniquely predict higher TSD PM SVRI. Our results also show that lower TSD PM SV can be predicted a priori in those individuals who have higher WASO and lower percent sleep the night before sleep loss and during the pre‐experimental (habitual) sleep phase. In addition, higher B2 WASO also significantly predicted higher B2 AM SVRI, which may indicate that sleep continuity the night before is related to the next morning's vascular resistance. B2 night sleep indices did not significantly predict blood pressure metrics consistent with some prior studies (Bigalke et al., 2024; Mezick et al., 2012), but in contrast with others (Abbott et al., 2019; Bommarito & Millar, 2024; Chang & Kang, 2021; Eiman et al., 2019; Fujikawa et al., 2009; Jansen et al., 2020; Liang et al., 2024; Massar et al., 2017; Mezick et al., 2014; Springall De Pablo & Lauderdale, 2024).

Our findings demonstrated that longer TSD AM and TSD PM LVET predicted later R1 sleep offset and longer R1 sleep duration, and longer TSD PM LVET predicted higher R1 WASO. In contrast, higher TSD AM and TSD PM HR predicted earlier R1 sleep offset. Similar to these results, we previously found a significant negative association between LVET and HR during TSD (Pasetes et al., 2023a). In a prior study, we found that individuals with cognitive vulnerability during TSD and psychological stress had longer LVET (Yamazaki, Rosendahl‐Garcia, et al., 2022) and that TSD increased LVET, indicating that longer LVET may be a harmful rather than an adaptive response (Pasetes et al., 2023b; Yamazaki, Rosendahl‐Garcia, et al., 2022). Therefore, longer LVET may be indicative of hyperarousal, which is reflected in higher WASO during the subsequent recovery night. Similarly, higher TSD AM and TSD PM HR may signify more stress (Bloomfield et al., 2024), which is reflected in earlier awakening during recovery sleep. Notably, we previously found that TSD AM and TSD PM LVET did not significantly differ; however, TSD AM HR was significantly lower than TSD PM HR (Yamazaki, Rosendahl‐Garcia, et al., 2022). Notably, the R1 sleep timing and duration data following TSD are representative of and similar to those in our prior studies using unrelated samples (Pasetes et al., 2023a, 2023b; Yamazaki, Antler, Lasek, et al., 2021). Little is known about the factors that influence recovery sleep after TSD; thus, future research should further examine the predictive relationships between CV measures during TSD and psychological stress and sleep metrics during recovery.

We found that B2 percent sleep was significantly and negatively correlated with B2 WASO, which likely explains why we found B2 percent sleep and B2 WASO yielded opposite predictive relationships with TSD PM SVRI and TSD PM SV. In addition, B2 sleep efficiency, which was positively related to B2 percent sleep, showed the same predictive relationships with TSD PM SVRI and TSD PM SV, but these did not reach statistical significance (*p* = 0.053–0.055). In addition, R1 sleep duration, R1 sleep offset, and R1 WASO were all significantly and positively correlated with each other, reflected in their similar predictive relationships with TSD AM and TSD PM LVET. Hence, R1 sleep efficiency and R1 percent sleep, which are negatively correlated with these R1 sleep measures, may show significant negative predictive relationships with TSD AM and TSD PM LVET in a larger sample. Future studies should investigate these relationships further to inform biomarker research in this area.

There are established sex differences in sleep measures (Johnson et al., 2024; Lok et al., 2024; Mulè et al., 2021; Roberts et al., 2022) as well as in CV measures (Drury et al., 2024; Lin et al., 2023; Quer et al., 2020; Wooten et al., 2021). We found that the predictive bidirectional relationships between sleep and CV metrics were significant and explained more of the variance in females. While this is the first examination of bidirectional relationships between baseline and recovery sleep and CV measures during TSD and psychological stress in females and males, other studies generally also found that females showed stronger associations between sleep metrics and CV measures in conditions without sleep loss or psychological stress (Forshaw et al., 2024; Hoopes et al., 2021; Killick et al., 2023; Liang et al., 2024; Makarem et al., 2019). Stronger bidirectional predictions in females are in line with the more robust association between poorer sleep health metrics and higher risk for cardiovascular disease and dysfunction in females (Nikbakhtian et al., 2021; Wright et al., 2023), and the well‐established relationship between female reproductive hormones and sleep (Baker et al., 2012; Wright et al., 2023).

We have previously shown that both actigraphic sleep and CV indices are stable across time during sleep loss (Pasetes et al., 2023a; Pasetes & Goel, 2024); thus, we assume that the predictive bidirectional relationships between these metrics would be maintained and remain significant with repeated exposures to TSD. Therefore, our results have implications for populations who are at risk for or have a CV event or disease such as hypertension, coronary heart disease, congestive heart failure, heart attack, or myocardial infarction (Diao et al., 2023; Fan et al., 2021; Haghayegh et al., 2023; Killick et al., 2023; Kogon et al., 2024; Nikbakhtian et al., 2021; Ujma & Bódizs, 2024; Yan, Li, et al., 2021; Yan, Wu, et al., 2021; Yeung et al., 2023).

Our study has a few limitations. Since we had a smaller sample size, we did not add age or other factors into our linear regression analyses, and we did not correct for multiple comparisons for our main findings. Therefore, future studies should verify the results of this study using larger sample sizes. Since the mental stress task was only conducted in the afternoon, it is unclear whether our significant TSD PM results were due to a time‐of‐day effect or due to psychological stress. Moreover, we did not collect B2 PM SV and SVRI measures; thus, there is no appropriate comparison to subtract TSD PM SV and SVRI measures from B2 AM SV and SVRI measures to determine whether B2 sleep is predictive of TSD PM‐evoked changes. Furthermore, since our study was conducted in isolation and in highly controlled conditions in order to simulate a long‐duration space flight environment, our results may not be generalizable to situations that do not involve such settings (Le Roy et al., 2023). In addition, we used sleep midpoint as a proxy of circadian phase, but we do not have any other measures of circadian phase. Of note, in these studies, we could not examine or control for the menstrual cycle. Finally, the velocity time integral of the continuous wave form of the ascending aorta was used as a surrogate measure for left ventricular outflow tract velocity time integral in our study.

Our novel results found that actigraphic sleep metrics the night before TSD uniquely predicted SV and SVRI during TSD and psychological stress (TSD PM) in healthy adults. Moreover, this is the first demonstration that CV indices during TSD and psychological stress are reflected in the duration, quality, and timing of actigraphic sleep during the subsequent recovery night. Thus, WASO and percent sleep during fully rested conditions may serve as biomarkers to predict individual differences in CV measures during TSD and psychological stress, and similarly, LVET and HR during TSD may be biomarkers for determining individual differences in recovery sleep. Notably, the echocardiographic CV measures assessed in this study have been rarely used in sleep loss studies; thus, our results add important information to the literature on these biomarkers and predictors. Overall, the predictive bidirectional relationships between sleep metrics and CV measures were significant and explained more of the variance in females. Our results underscore the critical relationships and mechanisms between metrics of sleep health and cardiovascular health.

## AUTHOR CONTRIBUTIONS

Conceptualization, N.G.; methodology, N.G.; validation, N.G., L.N.P., and K.M.R‐G.; formal analysis, L.N.P. and K.M.R‐G.; investigation, N.G.; resources, N.G.; data curation, N.G.; writing—original draft preparation, L.N.P., N.G., and K.M.R‐G.; writing—review and editing, N.G., L.N.P., and K.M.R‐G.; visualization, L.N.P. and N.G.; supervision, N.G.; project administration, N.G.; funding acquisition, N.G. All authors have read and agreed to the submitted version of the manuscript.

## FUNDING INFORMATION

This research was funded by the National Aeronautics and Space Administration (NASA) [grant numbers NNX14AN49G and 80NSSC20K0243 (to N.G.)]. This work was also partially supported by the National Institutes of Health [grant number NIH R01DK117488 (to N.G.)].

## CONFLICT OF INTEREST STATEMENT

The authors declare that the research was conducted in the absence of any commercial or financial relationships that could be construed as a potential conflict of interest.

## ETHICS STATEMENT

The study was approved by the Institutional Review Boards of NASA, who had primary oversight, and by the University of Pennsylvania, and all protocol methods were carried out in accordance with approved guidelines and regulations. Prior to inclusion in the study, participants provided written informed consent, which was in accordance with the Declaration of Helsinki.

## Data Availability

The data generated during and/or analyzed during the current study are available from the corresponding author upon reasonable request.

## References

[phy270374-bib-0001] Abbott, S. M. , Weng, J. , Reid, K. J. , Daviglus, M. L. , Gallo, L. C. , Loredo, J. S. , Nyenhuis, S. M. , Ramos, A. R. , Shah, N. A. , Sotres‐Alvarez, D. , Patel, S. R. , & Zee, P. C. (2019). Sleep timing, stability, and BP in the Sueño ancillary study of the Hispanic community health study/study of Latinos. Chest, 155, 60–68. 10.1016/j.chest.2018.09.018 30300651 PMC6344384

[phy270374-bib-0002] Abeln, V. , Fomina, E. , Popova, J. , Braunsmann, L. , Koschate, J. , Möller, F. , Fedyay, S. O. , Vassilieva, G. Y. , Schneider, S. , Strüder, H. K. , & Klein, T. (2022). Chronic, acute and protocol‐dependent effects of exercise on psycho‐physiological health during long‐term isolation and confinement. BMC Neuroscience, 23, 41. 10.1186/s12868-022-00723-x 35773633 PMC9244384

[phy270374-bib-0003] Allen, A. P. , Kennedy, P. J. , Cryan, J. F. , Dinan, T. G. , & Clarke, G. (2014). Biological and psychological markers of stress in humans: Focus on the Trier Social Stress Test. Neuroscience and Biobehavioral Reviews, 38, 94–124. 10.1016/j.neubiorev.2013.11.005 24239854

[phy270374-bib-0004] Arbeille, P. , & Herault, S. (1998). Cardiovascular echographic and Doppler parameters for the assessment of orthostatic intolerance. European Journal of Ultrasound, 7, 53–71. 10.1016/s0929-8266(98)00019-6 9614291

[phy270374-bib-0005] Baker, F. C. , Sassoon, S. A. , Kahan, T. , Palaniappan, L. , Nicholas, C. L. , Trinder, J. , & Colrain, I. M. (2012). Perceived poor sleep quality in the absence of polysomnographic sleep disturbance in women with severe premenstrual syndrome. Journal of Sleep Research, 21, 535–545. 10.1111/j.1365-2869.2012.01007.x 22417163 PMC3376683

[phy270374-bib-0006] Bigalke, J. A. , Greenlund, I. M. , Bigalke, J. R. , & Carter, J. R. (2024). Actigraphy‐based sleep and muscle sympathetic nerve activity in humans. American Journal of Physiology. Regulatory, Integrative and Comparative Physiology, 327, R145–R151. 10.1152/ajpregu.00113.2024 38842513 PMC11444507

[phy270374-bib-0007] Bloomfield, L. S. P. , Fudolig, M. I. , Kim, J. , Llorin, J. , Lovato, J. L. , McGinnis, E. W. , McGinnis, R. S. , Price, M. , Ricketts, T. H. , Dodds, P. S. , Stanton, K. , & Danforth, C. M. (2024). Predicting stress in first‐year college students using sleep data from wearable devices. PLOS Digital Health, 3, e0000473. 10.1371/journal.pdig.0000473 38602898 PMC11008774

[phy270374-bib-0008] Bommarito, J. C. , & Millar, P. J. (2024). Effects of aerobic exercise on ambulatory blood pressure responses to acute partial sleep deprivation: Impact of chronotype and sleep quality. American Journal of Physiology. Heart and Circulatory Physiology, 326, H291–H301. 10.1152/ajpheart.00441.2023 38038716

[phy270374-bib-0009] Bozer, Ö. , Kaya, O. , Öztürk, G. , Bulut, E. , Zorkun, C. , & Öztürk, L. (2021). Blood pressure, autonomic stress, and inflammatory markers during sleep deprivation and recovery in healthy men. Anatolian Journal of Cardiology, 25, 407–413. 10.14744/AnatolJCardiol.2020.42205 34100728 PMC8210936

[phy270374-bib-0010] Cappuccio, F. P. , Cooper, D. , D'Elia, L. , Strazzullo, P. , & Miller, M. A. (2011). Sleep duration predicts cardiovascular outcomes: A systematic review and meta‐analysis of prospective studies. European Heart Journal, 32, 1484–1492. 10.1093/eurheartj/ehr007 21300732

[phy270374-bib-0011] Cattermole, G. N. , Leung, P. M. , Ho, G. Y. , Lau, P. W. , Chan, C. P. , Chan, S. S. , Smith, B. E. , Graham, C. A. , & Rainer, T. H. (2017). The normal ranges of cardiovascular parameters measured using the ultrasonic cardiac output monitor. Physiological Reports, 5, e13195. 10.14814/phy2.13195 28320891 PMC5371563

[phy270374-bib-0012] Chang, S. W. , & Kang, J. W. (2021). Association between sleep time and blood pressure in Korean adolescents: Cross‐sectional analysis of KNHANES VII. Children (Basel), 8, 1202. 10.3390/children8121202 34943398 PMC8700331

[phy270374-bib-0014] Dennis, L. E. , Wohl, R. J. , Selame, L. A. , & Goel, N. (2017). Healthy adults display long‐term trait‐like neurobehavioral resilience and vulnerability to sleep loss. Scientific Reports, 7, 14889. 10.1038/s41598-017-14006-7 29097703 PMC5668275

[phy270374-bib-0015] Diao, T. , Liu, K. , Wang, Q. , Lyu, J. , Zhou, L. , Yuan, Y. , Wang, H. , Yang, H. , Wu, T. , & Zhang, X. (2023). Bedtime, sleep pattern, and incident cardiovascular disease in middle‐aged and older Chinese adults: The Dongfeng‐Tongji cohort study. Sleep Medicine, 110, 82–88. 10.1016/j.sleep.2023.08.002 37544277

[phy270374-bib-0016] Doyle, C. Y. , Ruiz, J. M. , Taylor, D. J. , Smyth, J. W. , Flores, M. , Dietch, J. R. , Ahn, C. , Allison, M. , Smith, T. W. , & Uchino, B. N. (2019). Associations between objective sleep and ambulatory blood pressure in a community sample. Psychosomatic Medicine, 81, 545–556. 10.1097/PSY.0000000000000711 31083055 PMC6607429

[phy270374-bib-0017] Drury, E. R. , Wu, J. , Gigliotti, J. C. , & Le, T. H. (2024). Sex differences in blood pressure regulation and hypertension: Renal, hemodynamic, and hormonal mechanisms. Physiological Reviews, 104, 199–251. 10.1152/physrev.00041.2022 37477622 PMC11281816

[phy270374-bib-0018] Eiman, M. N. , Pomeroy, J. M. L. , & Weinstein, A. A. (2019). Relationship of actigraphy‐assessed sleep efficiency and sleep duration to reactivity to stress. Sleep Science, 12, 257–264. 10.5935/1984-0063.20190090 32318246 PMC7159077

[phy270374-bib-0019] Ekstedt, M. , Akerstedt, T. , & Söderström, M. (2004). Microarousals during sleep are associated with increased levels of lipids, cortisol, and blood pressure. Psychosomatic Medicine, 66, 925–931. 10.1097/01.psy.0000145821.25453.f7 15564359

[phy270374-bib-0020] Fan, Y. , Wu, Y. , Peng, Y. , Zhao, B. , Yang, J. , Bai, L. , Ma, X. , & Yan, B. (2021). Sleeping late increases the risk of myocardial infarction in the middle‐aged and older populations. Frontiers in Cardiovascular Medicine, 8, 709468. 10.3389/fcvm.2021.709468 34631815 PMC8498336

[phy270374-bib-0021] Fonseca, L. M. , Finlay, M. G. , Chaytor, N. S. , Morimoto, N. G. , Buchwald, D. , Van Dongen, H. P. A. , Quan, S. F. , & Suchy‐Dicey, A. (2024). Mid‐life sleep is associated with cognitive performance later in life in aging American Indians: Data from the Strong Heart Study. Frontiers in Aging Neuroscience, 16, 1346807. 10.3389/fnagi.2024.1346807 38903901 PMC11188442

[phy270374-bib-0022] Forshaw, P. E. , Correia, A. T. , Roden, L. C. , Lambert, E. V. , Layden, B. T. , Reutrakul, S. , Crowley, S. J. , Luke, A. , Dugas, L. R. , & Rae, D. E. (2024). Sex‐specific associations between self‐reported sleep characteristics and 10‐year cardiovascular disease risk in men and women of African descent living in a low socioeconomic status environment. Sleep Epidemiology, 4, 100091. 10.1016/j.sleepe.2024.100091 39801800 PMC11720418

[phy270374-bib-0023] Fujikawa, T. , Tochikubo, O. , Kura, N. , & Umemura, S. (2009). Factors related to elevated 24‐h blood pressure in young adults. Clinical and Experimental Hypertension, 31, 705–712. 10.3109/10641960903254422 20001463

[phy270374-bib-0024] Haghayegh, S. , Strohmaier, S. , Hamaya, R. , Eliassen, A. H. , Willett, W. C. , Rimm, E. B. , & Schernhammer, E. S. (2023). Sleeping difficulties, sleep duration, and risk of hypertension in women. Hypertension, 80, 2407–2414. 10.1161/HYPERTENSIONAHA.123.21350 37721046 PMC10591959

[phy270374-bib-0025] Helminen, E. C. , Morton, M. L. , Wang, Q. , & Felver, J. C. (2021). Stress reactivity to the Trier Social Stress Test in traditional and virtual environments: A meta‐analytic comparison. Psychosomatic Medicine, 83, 200–211. 10.1097/PSY.0000000000000918 33534392

[phy270374-bib-0026] Hoopes, E. K. , Patterson, F. , Berube, F. R. , D'Agata, M. N. , Brewer, B. , Malone, S. K. , Farquhar, W. B. , & Witman, M. A. (2021). Actigraphy‐derived rest‐‐activity rhythms are associated with nocturnal blood pressure in young women. Journal of Hypertension, 39, 2413–2421. 10.1097/HJH.0000000000002966 34387571 PMC8570977

[phy270374-bib-0027] Ihlen, H. , Amlie, J. P. , Dale, J. , Forfang, K. , Nitter‐Hauge, S. , Otterstad, J. E. , Simonsen, S. , & Myhre, E. (1984). Determination of cardiac output by Doppler echocardiography. British Heart Journal, 51, 54–60. 10.1136/hrt.51.1.54 6689921 PMC482313

[phy270374-bib-0028] James, J. E. , Douglas Gregg, M. E. , Matyas, T. A. , Hughes, B. M. , & Howard, S. (2012). Stress reactivity and the Hemodynamic Profile‐Compensation Deficit (HP‐CD) Model of blood pressure regulation. Biological Psychology, 90, 161–170. 10.1016/j.biopsycho.2012.02.021 22414744

[phy270374-bib-0029] Jansen, E. C. , Dunietz, G. L. , Matos‐Moreno, A. , Solano, M. , Lazcano‐Ponce, E. , & Sanchez‐Zamorano, L. M. (2020). Bedtimes and blood pressure: A prospective cohort study of Mexican adolescents. American Journal of Hypertension, 33, 269–277. 10.1093/ajh/hpz191 31840156 PMC7069344

[phy270374-bib-0030] Jayasinghe, S. U. , Torres, S. J. , Hussein, M. , Fraser, S. F. , Lambert, G. W. , & Turner, A. I. (2017). Fitter women did not have attenuated hemodynamic responses to psychological stress compared with age‐matched women with lower levels of fitness. PLoS One, 12, e0169746. 10.1371/journal.pone.0169746 28081200 PMC5231401

[phy270374-bib-0031] Johnson, D. A. , Wallace, D. A. , & Ward, L. (2024). Racial/ethnic and sex differences in the association between light at night and actigraphy‐measured sleep duration in adults: NHANES 2011‐2014. Sleep Health, 10, S184–S190. 10.1016/j.sleh.2023.09.011 37951773 PMC11031299

[phy270374-bib-0032] Kato, M. , Phillips, B. G. , Sigurdsson, G. , Narkiewicz, K. , Pesek, C. A. , & Somers, V. K. (2000). Effects of sleep deprivation on neural circulatory control. Hypertension, 35, 1173–1175. 10.1161/01.hyp.35.5.1173 10818083

[phy270374-bib-0033] Kelly, O. , Matheson, K. , Martinez, A. , Merali, Z. , & Anisman, H. (2007). Psychosocial stress evoked by a virtual audience: Relation to neuroendocrine activity. CyberPsychology & Behavior, 10, 655–662. 10.1089/cpb.2007.9973 17927533

[phy270374-bib-0034] Killick, R. , Stranks, L. , & Hoyos, C. M. (2023). Sleep deficiency and cardiometabolic disease. Sleep Medicine Clinics, 18, 331–347. 10.1016/j.jsmc.2023.05.012 37532373

[phy270374-bib-0035] Kirschbaum, C. , Pirke, K. M. , & Hellhammer, D. H. (1993). The ‘Trier Social Stress Test’—A tool for investigating psychobiological stress responses in a laboratory setting. Neuropsychobiology, 28, 76–81. 10.1159/000119004 8255414

[phy270374-bib-0036] Klabunde, R. E. (2012). Cardiovascular physiology concepts. Wolters Kluwer Health/Lippincott Williams & Wilkins.

[phy270374-bib-0037] Kogon, A. J. , Maqsood, A. M. , LoGuidice, J. , Amaral, S. , Meyers, K. , & Mitchell, J. A. (2024). Sleep duration and blood pressure in youth referred for elevated blood pressure evaluation. Pediatrics, 154, e2023062940. 10.1542/peds.2023-062940 38887814 PMC12354344

[phy270374-bib-0038] Kuetting, D. L. , Feisst, A. , Sprinkart, A. M. , Homsi, R. , Luetkens, J. , Thomas, D. , Schild, H. H. , & Dabir, D. (2019). Effects of a 24‐hr‐shift‐related short‐term sleep deprivation on cardiac function: A cardiac magnetic resonance‐based study. Journal of Sleep Research, 28, e12665. 10.1111/jsr.12665 29411477

[phy270374-bib-0039] Le Roy, B. , Martin‐Krumm, C. , Pinol, N. , Dutheil, F. , & Trousselard, M. (2023). Human challenges to adaptation to extreme professional environments: A systematic review. Neuroscience and Biobehavioral Reviews, 146, 105054. 10.1016/j.neubiorev.2023.105054 36682426

[phy270374-bib-0040] Liang, X. , He, X. , Liu, Q. , Ren, Y. , Xu, S. , Chen, L. , Wang, F. , Bi, Y. , & Peng, Z. (2024). The impact of dietary and sleep rhythms on blood pressure in children and adolescents: A cross‐sectional study. Hypertension Research, 47, 649–662. 10.1038/s41440-023-01493-7 37919430

[phy270374-bib-0041] Liew, S. C. , & Aung, T. (2021). Sleep deprivation and its association with diseases—A review. Sleep Medicine, 77, 192–204. 10.1016/j.sleep.2020.07.048 32951993

[phy270374-bib-0042] Lin, H. , Kwan, A. C. , Castro‐Diehl, C. , Short, M. I. , Xanthakis, V. , Yola, I. M. , Salto, G. , Mitchell, G. F. , Larson, M. G. , Vasan, R. S. , & Cheng, S. (2023). Sex‐specific differences in the genetic and environmental effects on cardiac phenotypic variation assessed by echocardiography. Scientific Reports, 13, 5786. 10.1038/s41598-023-32577-6 37031215 PMC10082757

[phy270374-bib-0043] Liu, H. , & Chen, A. (2019). Roles of sleep deprivation in cardiovascular dysfunctions. Life Sciences, 219, 231–237. 10.1016/j.lfs.2019.01.006 30630005

[phy270374-bib-0044] Lloyd‐Jones, D. M. , Allen, N. B. , Anderson, C. A. M. , Black, T. , Brewer, L. C. , Foraker, R. E. , Grandner, M. A. , Lavretsky, H. , Perak, A. M. , Sharma, G. , & Rosamond, W. (2022). Life's essential 8: Updating and enhancing the American Heart Association's construct of cardiovascular health: A presidential advisory from the American Heart Association. Circulation, 146, e18–e43. 10.1161/CIR.0000000000001078 35766027 PMC10503546

[phy270374-bib-0045] Lok, R. , Qian, J. , & Chellappa, S. L. (2024). Sex differences in sleep, circadian rhythms, and metabolism: Implications for precision medicine. Sleep Medicine Reviews, 75, 101926. 10.1016/j.smrv.2024.101926 38564856

[phy270374-bib-0046] Lü, W. , Hughes, B. M. , Howard, S. , & James, J. E. (2018). Sleep restriction undermines cardiovascular adaptation during stress, contingent on emotional stability. Biological Psychology, 132, 125–132. 10.1016/j.biopsycho.2017.11.013 29246812

[phy270374-bib-0047] Makarem, N. , Shechter, A. , Carnethon, M. R. , Mullington, J. M. , Hall, M. H. , & Abdalla, M. (2019). Sleep duration and blood pressure: Recent advances and future directions. Current Hypertension Reports, 21, 33. 10.1007/s11906-019-0938-7 30953237 PMC10239254

[phy270374-bib-0048] Massar, S. A. A. , Liu, J. C. J. , Mohammad, N. B. , & Chee, M. W. L. (2017). Poor habitual sleep efficiency is associated with increased cardiovascular and cortisol stress reactivity in men. Psychoneuroendocrinology, 81, 151–156. 10.1016/j.psyneuen.2017.04.013 28482312

[phy270374-bib-0049] McLennan, F. M. , Haites, N. E. , Mackenzie, J. D. , Daniel, M. K. , & Rawles, J. M. (1986). Reproducibility of linear cardiac output measurement by Doppler ultrasound alone. British Heart Journal, 55, 25–31. 10.1136/hrt.55.1.25 3511929 PMC1232064

[phy270374-bib-0050] Mezick, E. J. , Hall, M. , & Matthews, K. A. (2012). Sleep duration and ambulatory blood pressure in black and white adolescents. Hypertension, 59, 747–752. 10.1161/HYPERTENSIONAHA.111.184770 22275538 PMC3314491

[phy270374-bib-0051] Mezick, E. J. , Matthews, K. A. , Hall, M. H. , Jennings, R. J. , & Kamarck, T. W. (2014). Sleep duration and cardiovascular responses to stress in undergraduate men. Psychophysiology, 51, 88–96. 10.1111/psyp.12144 24016263 PMC3883723

[phy270374-bib-0052] Mikulski, T. , Tomczak, A. , Lejk, P. , & Klukowski, K. (2006). Influence of ultra long exercise and sleep deprivation on physical performance of healthy men. Medicina Sportiva, 10, 98–101.

[phy270374-bib-0053] Moreno‐Villanueva, M. , von Scheven, G. , Feiveson, A. , Bürkle, A. , Wu, H. , & Goel, N. (2018). The degree of radiation‐induced DNA strand breaks is altered by acute sleep deprivation and psychological stress and is associated with cognitive performance in humans. Sleep, 41, 1–9. 10.1093/sleep/zsy067 29596659

[phy270374-bib-0054] Mulè, A. , Bruno, E. , Pasanisi, P. , Galasso, L. , Castelli, L. , Caumo, A. , Esposito, F. , Roveda, E. , & Montaruli, A. (2021). Sex differences in rest‐activity circadian rhythm in patients with metabolic syndrome. Frontiers in Physiology, 12, 641461. 10.3389/fphys.2021.641461 33815145 PMC8013705

[phy270374-bib-0055] Mullington, J. M. , Haack, M. , Toth, M. , Serrador, J. M. , & Meier‐Ewert, H. K. (2009). Cardiovascular, inflammatory, and metabolic consequences of sleep deprivation. Progress in Cardiovascular Diseases, 51, 294–302. 10.1016/j.pcad.2008.10.003 19110131 PMC3403737

[phy270374-bib-0056] Nikbakhtian, S. , Reed, A. B. , Obika, B. D. , Morelli, D. , Cunningham, A. C. , Aral, M. , & Plans, D. (2021). Accelerometer‐derived sleep onset timing and cardiovascular disease incidence: A UK Biobank cohort study. European Heart Journal ‐ Digital Health, 2, 658–666. 10.1093/ehjdh/ztab088 36713092 PMC9708010

[phy270374-bib-0057] Norsk, P. , Asmar, A. , Damgaard, M. , & Christensen, N. J. (2015). Fluid shifts, vasodilation and ambulatory blood pressure reduction during long duration spaceflight. Journal of Physiology, 593, 573–584. 10.1113/jphysiol.2014.284869 25774397 PMC4324706

[phy270374-bib-0058] Orme, S. , Ralph, S. G. , Birchall, A. , Lawson‐Matthew, P. , McLean, K. , & Channer, K. S. (1999). The normal range for inter‐arm differences in blood pressure. Age and Ageing, 28, 537–542. 10.1093/ageing/28.6.537 10604505

[phy270374-bib-0059] Pasetes, L. N. , & Goel, N. (2024). Short‐term and long‐term phenotypic stability of actigraphic sleep metrics involving repeated sleep loss and recovery. Journal of Sleep Research, 33, e14149. 10.1111/jsr.14149 38284151 PMC11284248

[phy270374-bib-0060] Pasetes, L. N. , Rosendahl‐Garcia, K. M. , & Goel, N. (2023a). Cardiovascular measures display robust phenotypic stability across long‐duration intervals involving repeated sleep deprivation and recovery. Frontiers in Neuroscience, 17, 1201637. 10.3389/fnins.2023.1201637 37547137 PMC10397520

[phy270374-bib-0061] Pasetes, L. N. , Rosendahl‐Garcia, K. M. , & Goel, N. (2023b). Impact of bimonthly repeated total sleep deprivation and recovery sleep on cardiovascular indices. Physiological Reports, 11, e15841. 10.14814/phy2.15841 37849046 PMC10582224

[phy270374-bib-0062] Quer, G. , Gouda, P. , Galarnyk, M. , Topol, E. J. , & Steinhubl, S. R. (2020). Inter‐ and intraindividual variability in daily resting heart rate and its associations with age, sex, sleep, BMI, and time of year: Retrospective, longitudinal cohort study of 92,457 adults. PLoS One, 15, e0227709. 10.1371/journal.pone.0227709 32023264 PMC7001906

[phy270374-bib-0063] Roberts, S. S. H. , Main, L. C. , Condo, D. , Carr, A. , Jardine, W. , Urwin, C. , Convit, L. , Rahman, S. S. , & Snipe, R. M. J. (2022). Sex differences among endurance athletes in the pre‐race relationships between sleep, and perceived stress and recovery. Journal of Sports Sciences, 40, 1542–1551. 10.1080/02640414.2022.2091345 35767576

[phy270374-bib-0064] Ruiz, A. S. , Peralta‐Ramirez, M. I. , Garcia‐Rios, M. C. , Muñoz, M. A. , Navarrete‐Navarrete, N. , & Blazquez‐Ortiz, A. (2010). Adaptation of the Trier Social Stress Test to virtual reality: Psycho‐physiological and neuroendocrine modulation. Journal of Cyber Therapy and Rehabilitation, 3, 405–415.

[phy270374-bib-0065] Saveko, A. , Brykov, V. , Kitov, V. , Shpakov, A. , & Tomilovskaya, E. (2022). Adaptation in gait to lunar and Martian gravity unloading during long‐term isolation in the ground‐based space station model. Frontiers in Human Neuroscience, 15, 742664. 10.3389/fnhum.2021.742664 35095445 PMC8790089

[phy270374-bib-0066] Sekiguchi, Y. , Adams, W. M. , Benjamin, C. L. , Curtis, R. M. , Giersch, G. E. W. , & Casa, D. J. (2019). Relationships between resting heart rate, heart rate variability and sleep characteristics among female collegiate cross‐country athletes. Journal of Sleep Research, 28, e12836. 10.1111/jsr.12836 30843295

[phy270374-bib-0067] Shaffer, F. , & Ginsberg, J. P. (2017). An overview of heart rate variability metrics and norms. Frontiers in Public Health, 5, 258. 10.3389/fpubh.2017.00258 29034226 PMC5624990

[phy270374-bib-0013] Springall De Pablo, M. , & Lauderdale, D. S. (2024). Associations of actigraph sleep characteristics with blood pressure among older adults. Sleep Health, 10, 455–461. 10.1016/j.sleh.2024.04.002 38906803 PMC11500670

[phy270374-bib-0068] Tobaldini, E. , Costantino, G. , Solbiati, M. , Cogliati, C. , Kara, T. , Nobili, L. , & Montano, N. (2017). Sleep, sleep deprivation, autonomic nervous system and cardiovascular diseases. Neuroscience and Biobehavioral Reviews, 74, 321–329. 10.1016/j.neubiorev.2016.07.004 27397854

[phy270374-bib-0069] Turner, A. I. , Smyth, N. , Hall, S. J. , Torres, S. J. , Hussein, M. , Jayasinghe, S. U. , Ball, K. , & Clow, A. J. (2020). Psychological stress reactivity and future health and disease outcomes: A systematic review of prospective evidence. Psychoneuroendocrinology, 114, 104599. 10.1016/j.psyneuen.2020.104599 32045797

[phy270374-bib-0070] Ujma, P. P. , & Bódizs, R. (2024). Sleep alterations as a function of 88 health indicators. BMC Medicine, 22, 134. 10.1186/s12916-024-03358-3 38519958 PMC10960465

[phy270374-bib-0071] Wooten, S. V. , Moestl, S. , Chilibeck, P. , Alvero Cruz, J. R. , Mittag, U. , Tank, J. , Tanaka, H. , Rittweger, J. , & Hoffmann, F. (2021). Age‐ and sex‐differences in cardiac characteristics determined by echocardiography in masters athletes. Frontiers in Physiology, 11, 630148. 10.3389/fphys.2020.630148 33536945 PMC7848176

[phy270374-bib-0072] Wright, C. J. , Milosavljevic, S. , & Pocivavsek, A. (2023). The stress of losing sleep: Sex‐specific neurobiological outcomes. Neurobiology of Stress, 24, 100543. 10.1016/j.ynstr.2023.100543 37252645 PMC10209346

[phy270374-bib-0076] Yamazaki, E. M. , & Goel, N. (2020). Robust stability of trait‐like vulnerability or resilience to common types of sleep deprivation in a large sample of adults. Sleep, 43, zsz292. 10.1093/sleep/zsz292 31784748 PMC8152927

[phy270374-bib-0073] Yamazaki, E. M. , Antler, C. A. , Casale, C. E. , MacMullen, L. E. , Ecker, A. J. , & Goel, N. (2021). Cortisol and C‐reactive protein vary during sleep loss and recovery but are not markers of neurobehavioral resilience. Frontiers in Physiology, 12, 782860. 10.3389/fphys.2021.782860 34912243 PMC8667577

[phy270374-bib-0074] Yamazaki, E. M. , Antler, C. A. , Lasek, C. R. , & Goel, N. (2021). Residual, differential neurobehavioral deficits linger after multiple recovery nights following chronic sleep restriction or acute total sleep deprivation. Sleep, 44, zsaa224. 10.1093/sleep/zsaa224 33274389 PMC8274462

[phy270374-bib-0075] Yamazaki, E. M. , Casale, C. E. , Brieva, T. E. , Antler, C. A. , & Goel, N. (2022). Concordance of multiple methods to define resiliency and vulnerability to sleep loss depends on Psychomotor Vigilance Test metric. Sleep, 45, zsab249. 10.1093/sleep/zsab249 34624897 PMC8754491

[phy270374-bib-0077] Yamazaki, E. M. , Rosendahl‐Garcia, K. M. , Casale, C. E. , MacMullen, L. E. , Ecker, A. J. , Kirkpatrick, J. N. , & Goel, N. (2022). Left ventricular ejection time measured by echocardiography differentiates neurobehavioral resilience and vulnerability to sleep loss and stress. Frontiers in Physiology, 12, 795321. 10.3389/fphys.2021.795321 35087419 PMC8787291

[phy270374-bib-0078] Yan, B. , Li, R. , Li, J. , Jin, X. , Gao, F. , Gao, Y. , Ren, J. , Zhang, J. , Wang, X. , & Wang, G. (2021). Sleep timing may predict congestive heart failure: A community‐based cohort study. Journal of the American Heart Association, 10, e018385. 10.1161/JAHA.120.018385 33666090 PMC8174199

[phy270374-bib-0079] Yan, B. , Wu, Y. , Fan, X. , Lu, Q. , Ma, X. , & Bai, L. (2021). Sleep fragmentation and incidence of congestive heart failure: The Sleep Heart Health Study. Journal of Clinical Sleep Medicine, 17, 1619–1625. 10.5664/jcsm.9270 33779541 PMC8656916

[phy270374-bib-0080] Yeung, C. H. C. , Bauer, C. , & Xiao, Q. (2023). Associations between actigraphy‐derived rest‐activity rhythm characteristics and hypertension in United States adults. Journal of Sleep Research, 32, e13854. 10.1111/jsr.13854 36807441

[phy270374-bib-0081] Yiallourou, S. R. , Maguire, G. P. , & Carrington, M. J. (2021). Sleep quantity and quality and cardiometabolic risk factors in indigenous Australians. Journal of Sleep Research, 30, e13067. 10.1111/jsr.13067 32526810

